# Arbeitsschutz bei der Versorgung von COVID-19-Patienten: Relevante Gesetze und Regelungen für das versorgende Personal

**DOI:** 10.1007/s00101-020-00828-0

**Published:** 2020-08-11

**Authors:** N. P. Mayr, S. Sernetz, F. Heitzer, M. Joner, P. Tassani-Prell

**Affiliations:** 1 Institut für Anästhesiologie, Deutsches Herzzentrum München des Freistaates Bayern, Technische Universität München, Lazarettstr. 36, 80636 München, Deutschland; 2 Deutsches Herzzentrum München des Freistaates Bayern, Technische Universität München, München, Deutschland; 3 Klinik für Herz- und Kreislauferkrankungen, Deutsches Herzzentrum München des Freistaates Bayern, Technische Universität München, München, Deutschland; 4 Partner Site Munich Heart Alliance, Deutsches Zentrum für Herz-Kreislauf-Forschung (DZHK) e. V., München, Deutschland

**Keywords:** Persönliche Schutzausrüstung, Intensivmedizin, Atemschutz, Berufskrankheit, Gefährdungsbeurteilung, Personal Protective Equipment, Intensive Care Medicine, Respiratory Protection, Occupational Disease, Risk assessment

## Abstract

Die intensivmedizinische Versorgung von COVID-19-Patienten stellt das eingesetzte Personal vor – bisher unbekannte – Herausforderungen. So kommen beispielsweise nun großflächig Schutzausrüstungen zum Einsatz, die ansonsten nur in ausgewählten Situationen verwendet wurden. Die Arbeit in einem solchen Umfeld ist unter dem Aspekt des Arbeitsschutzes anders zu bewerten als die sonstige Patientenversorgung. Auch in einer Pandemie bleiben die gesetzlichen Vorgaben gültig. Ziel dieser Arbeit ist es, einen Überblick über die aktuell relevanten Vorschriften und Regelungen darzulegen.

## Abstract (en)

The intensive medical care of COVID-19 patients presents the deployed personnel with as yet unknown challenges. For example, protective equipment is now being extensively used, which was otherwise only used in selected situations. Working in such an environment is to be evaluated differently under the aspect of occupational safety than other patient care, especially as more than 1900 suspected cases of a SARS-CoV-19 occupational disease were reported among healthcare workers in Germany. Even in a pandemic, the legal requirements remain valid and personal protective equipment (PPE) has to comply with given standards. The use of FFP3 masks is required in aerosol-forming situations, such as endotracheal intubation or bronchoscopy. In contrast to surgical face masks, there is a maximum wearing time for FFPs masks. Furthermore, in a pandemic there is a basic danger of PPE shortage and recycling of face masks is under discussion. Therefore, usage of non-EU certified PPE may come into effect but this has to follow the requirements defined by European regulations. The aim of this article is to provide an overview of the currently relevant rules and regulations in Germany.

## Hintergrund

Die aktuelle COVID-19-Pandemie stellt weltweit anästhesiologisches, notfallmedizinisches und intensivmedizinisches Personal vor unbekannte Herausforderungen. Neben der Vorhaltung und dem Aufbau von Intensivkapazitäten und Beatmungsgeräten ist der persönliche Schutz des Personals ein elementarer Bestandteil. In anderen Ländern (hier vornehmlich den USA und Großbritannien) ist ein Mangel an PSA-Ausstattung (PSA: persönliche Schutzausrüstung) offensichtlich. So findet man beispielsweise bei Twitter unter #ppnow zahlreiche Beispiele improvisierter Schutzausrüstung, wie Mülltüten oder Plastiktüten als Gesichtsschutz [31].

Die Wichtigkeit der PSA und der suffizienten Schutzausrüstung zeigt sich bei der endotrachealen Intubation besonders, wie zuletzt mittels eines simulierten Hustenstoßes gezeigt werden konnte [14]. Bis Ende April 2020 wurden über 1900 Verdachtsanzeigen bezüglich einer Berufskrankheit nach der Diagnosestellung einer SARS-CoV-2-Infektion bei Beschäftigten im Gesundheitswesen gestellt [28].

Ziel dieser Übersicht soll sein, die aktuelle Gesetzeslage bezüglich des Arbeitnehmerschutzes durch PSA darzustellen. Trotz einer landesweiten medizinischen Krisensituation sind die relevanten Gesetze und Verordnungen weiterhin als gültig anzusehen.

## Grundlage des Arbeitsschutzes in Deutschland

Als Arbeitsschutz werden alle Maßnahmen und Mittel verstanden, die den Beschäftigten vor arbeitsbedingten Sicherheits- und Gesundheitsgefährdungen bewahren sollen. Dazu gehören neben der Vermeidung von Arbeitsunfällen auch der Schutz vor akuten und chronischen Gesundheitsschäden. Die technischen und organisatorischen Maßnahmen durch eine adäquate PSA spielen hier eine grundlegende Rolle.

In Deutschland wird der Arbeitsschutz dual überwacht, zum einen durch die staatlichen Aufsichtsbehörden (Bundesländer, Regierungspräsidien, zuständige Landkreise) und zum anderen durch die Träger der gesetzlichen Unfallversicherung. Für nichtstaatliche Gesundheitseinrichtungen ist hierbei die gewerbliche Berufsgenossenschaft für Gesundheitsdienst und Wohlfahrtspflege (BGW) zuständig, für staatliche Gesundheitseinrichtungen die zuständige Unfallkasse der Länder.

Die wichtigste gesetzliche Grundlage des Arbeitsschutzes bildet das Arbeitsschutzgesetz (ArbSchG). Darin wird der Arbeitgeber verpflichtet, alle erforderlichen Maßnahmen einzuhalten und diese bei sich ändernden Gegebenheiten anzupassen (§ 3 ArbSchG) [10].

## Arbeitsschutz bei COVID-19

Im Fall von COVID-19 ist neben dem Beschluss 609 („Arbeitsschutz beim Auftreten einer nicht ausreichend impfpräventablen humanen Influenza“) [4] die staatliche Arbeitsschutzvorschrift „Verordnung über Sicherheit und Gesundheitsschutz bei Tätigkeiten mit Biologischen Arbeitsstoffen (BioStoffV)“ [11] und ihre konkretisierende „Technische Regel für Biologische Arbeitsstoffe 250 (TRBA 250)“ [2] in der 4. Änderung vom 02.05.2018 relevant. Diese Regel wird von dem Ausschuss für Biologische Arbeitsstoffe angepasst und im *Gemeinsamen Ministerialblatt* des Bundesministeriums für Arbeit und Soziales veröffentlicht. Damit bekommt diese Regel einen verpflichtenden Charakter. Anders als die TRBA 100, die sich auf Arbeiten in Laboratorien bezieht [3], zielt die TRBA 250 auf Tätigkeiten „in Bereichen des Gesundheitswesens und der Wohlfahrtspflege, in denen Menschen untersucht, behandelt oder gepflegt werden“.

Analog zum ArbSchG und zur BioStoffV ist gemäß TRBA 250 & TRBA 400 eine mindestens 2‑jährlich durchzuführende Gefährdungsbeurteilung mit Wirksamkeitsprüfung festgelegt. Eine Aktualisierung der Gefährdungsbeurteilung wird notwendig, wenn u. a. Veränderungen zu einer Gefährdung der Sicherheit und Gesundheit von Beschäftigten führen können. Dies ist beispielsweise bei dem Auftreten neuer Erreger – die eine besondere Schutzausrüstung benötigen – der Fall (TRBA 250, Abschn. 3.1.2) [5].

Für das Coronavirus SARS-CoV 2 erfolgte eine Einteilung gemäß BioStoffV in die Risikogruppe 3 (19.02.2020) [11] (Def.: Biostoffe, die eine schwere Krankheit beim Menschen hervorrufen und eine ernste Gefahr für Beschäftigte darstellen).

Neben weiterer Schutzausrüstung (z. B. Augenschutz, Hautschutz) ist für die Versorgung dieser Patienten mindestens eine Schutzmaske der Klasse FFP2 (FFP: Filtering Face Piece) vorgeschrieben. Sofern die Möglichkeit besteht, dass bei der Patientenversorgung Aerosole freigesetzt werden (z. B. Intubation, Bronchoskopie), ist eine FFP3-Schutzmaske zu tragen. Diese gelten, wie die andere Schutzkleidung als PSA im Sinne der europäischen Verordnung (EU) 2016/425 [26]. FFP-Masken gelten entsprechend dem DGUV-Grundsatz G26 [20] (DGUV: Deutsche Gesetzliche Unfallversicherung) bzw. der Arbeitsmedizinischen Regel 14.2 als „Atemschutzgerät der Gruppe 1 (bis 3 kg Gewicht und Atemwegswiderstand bis 5 mbar)“ [1]. Die Benutzung von Atemschutzgeräten bedeutet i. Allg. eine zusätzliche Belastung für den Träger.

**Figure 1:**
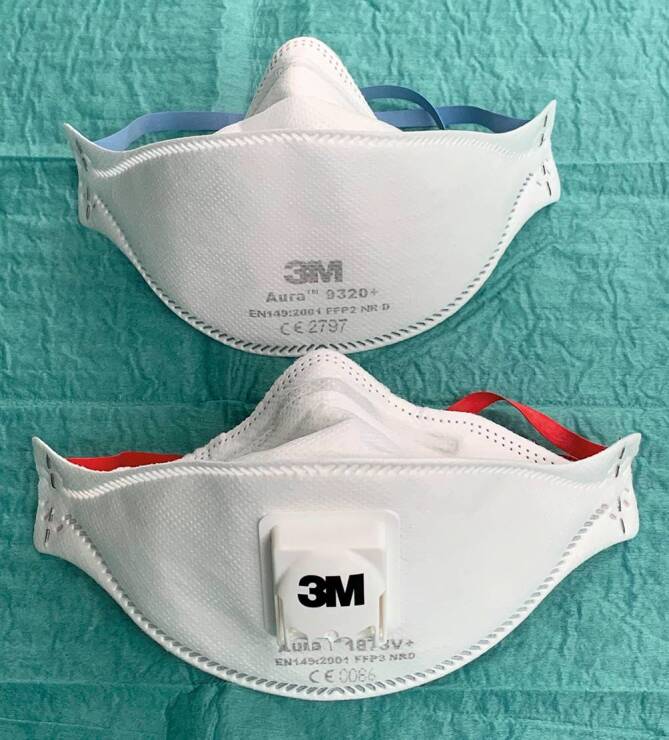

In Abhängigkeit von der betrieblichen Gefährdungsbeurteilung ist bei den Mitarbeitern eine entsprechende arbeitsmedizinische Vorsorge zu veranlassen bzw. anzubieten [20]. Es gilt derzeit die maximale Tragedauer bei Masken ohne Ausatemventil bei 75 min, während diese bei Masken mit Ausatemventil bei 120 min liegt (Abb. 1). Nach diesem Zeitraum ist bei beiden Maskentypen eine Erholungsdauer von 30 min vorgesehen. Die Einsätze pro Arbeitsschicht sind mit 5 (ohne Ausatemventil) und 3 (mit Ausatemventil) festgelegt. Die Arbeitsschichten pro Woche liegen bei 4 (ohne Ausatemventil) und 5 (mit Ausatemventil) (DGUV-Regel 112-190, Anhang 2, 5.1.3 & 5.1.4) [24].

Der Beschäftigte muss in den richtigen Gebrauch der PSA unterwiesen und ggf. geschult werden. Hierauf ist besonders zu achten, um in Bereichen erhöhter Infektionsgefährdung durch fehlerhafte Anwendung Kontaminationen und gesundheitliche Folgeschäden zu verhindern. Das Robert Koch-Institut hat beispielhafte Vorlagen für das korrekte An- und Ablegen der PSA auf seiner Website veröffentlicht [29].

Die FFP-Masken müssen gemäß (EU) 2016/425 mit einer „CE“-Kennzeichnung versehen sein [26]. Bedingt durch die derzeitigen Umstände empfiehlt die Bundesanstalt für Arbeitsschutz und Arbeitsmedizin (BAUA), ausländische Masken mit dem US-amerikanischen Mindeststandard NIOSH N95 (entspricht etwa FFP2) zu verwenden, sofern KEINE „CE-Masken“ zur Verfügung stehen. Alternative Kennzeichnungen sind in der Tab. 1 dargestellt [9]. Diese von der BAUA herausgegebene Übersicht hat nur einen unverbindlichen Informationscharakter und entspricht nicht dem Rechtscharakter einer Verordnung [8, 25].

**Table 1:** 

Land	Standard	Akzeptable Produktklassifizierung	Richtlinie
Australien	AS/NZS 1716:2012	P3 P2	AS/NZS 1715:2009
Brazil	ABNT/NBR 13698:2011	PFF3 PFF2	Fundacentro CDU 614.894
China	GB 2626-2006	KN100 KP100 KN95 KP95	GB/T 18664—2002
Europa	EN 149-2001	FFP3, FFP2	EN 529:2005
Japan	JMHLW-2000	DS/DL3 DS/DL2	JIS T8150: 2006
Korea	KMOEL-2017-64	Special 1st	KOSHA GUIDE H‑82-2015
Mexiko	NOM-116-2009	N100, P100, R100 N99, P99, R99 N95, P95, R95	NOM-116
US NIOSH Requirements	NIOSH approved 42 CFR 84	N100, P100, R100 N99, P99, R99 N95, P95, R95	OSHA 29CFR1910.134

Die Empfehlungen bezüglich eines ressourcenschonenden Einsatzes von Schutzausrüstung des Ad-hoc-Arbeitskreises „COVID-19“ des Ausschusses für biologische Arbeitsstoffe beim Bundesministerium für Arbeit und Soziales beziehen sich primär auf organisatorische Maßnahmen. Grundsätzlich werden partikelfiltrierende Halbmasken in 2 Gruppen eingeteilt: „NR“ („nonreusable“) und „R“ („reusable“). „NR“ bedeutet hierbei, dass der Mehrfachgebrauch „auf die Dauer einer Arbeitsschicht begrenzt ist“, während bei „R“-klassifizierten Masken eine Wiederbenutzung „über die Dauer einer Arbeitsschicht möglich ist“ (DGUV-Regel 112-190, 3.2.8.1) [18].

Derzeit wird in Deutschland die Wiederaufbereitung von „nonreusable“ FFP-Masken im Rahmen behördlich ausgerufener Notfallsituationen diskutiert. Dies könnte der Fall sein, wenn ein Mangel an Masken entsteht oder keine neuen Schutzmasken zur Verfügung stehen. Grundsätzlich ist eine Wiederaufbereitung von „nonreusable Masken“ kritisch zu sehen. Dieses Vorgehen entspricht nicht der originären Zweckbestimmung der PSA. Inwieweit sich etwaige Haftungsfragen ergeben, wenn wiederaufbereitete Masken ohne behördlich ausgerufene Notfallsituationen verwendet werden, bleibt zu diskutieren. Zu dem Thema „Wiederaufbereitung von Schutzmasken“ besteht behördlicherseits derzeit aktuell nur eine Vorlage des Bundesministeriums für Arbeit und Soziales und des Bundesministeriums für Gesundheit für den Krisenstab der Bundesregierung [12]. Bei CE-zertifizierten FFP-Masken erfolgt im Rahmen der Zulassung gemäß DIN EN 149 [21] eine Temperaturkonditionierung bei 70 °C über 24 h. Somit erscheint eine Wiederaufbereitung möglich. Bei Masken aus den USA, Kanada, Australien oder Japan erfolgt im Rahmen der jeweiligen länderspezifischen Zulassung nur eine Temperaturkonditionierung bei 38 °C. Daher ist vor einer möglichen Wiederaufbereitung erst eine Eingangsprüfung mit Temperaturkonditionierung von 70 °C über 24 h durchzuführen.

Das European Centre for Disease Prevention and Control (ECDC) hält eine Wiederverwendung von Schutzmasken nur in Ausnahmefällen für gerechtfertigt und hat hierzu im Juni 2020 eine Übersicht über die technisch möglichen Wiederaufbereitungsmöglichkeiten veröffentlicht [27]. In diesem „Technical Report“ werden v. a. auch die Nachteile der einzelnen Verfahren beleuchtet.

So erscheint die Dekontamination mittels Wasserstoffperoxiddampf als (bedingt) praktikabel. Nach 2 Wiederaufbereitungen wurden jedoch Verformungen mit einer potenziell veränderten Filtrierfähigkeit beschrieben. Weiterhin waren Wasserstoffperoxidrückstände noch Tage nach der Wiederaufbereitung nachweisbar. Als praktikable Alternative kommt eine Ausstattung des Personals mit 5 FFP-Masken in Betracht. Diese können dann – unter der Überlegung des Virenverfalls – wiederverwendet werden. Sichtbare Verunreinigungen oder Beschädigungen nach Wiederaufbereitung oder Lagerung stehen einer erneuten Verwendung entgegen.

Unsterile Einmalschutzkittel, die bei Barrieremaßnahmen und Isolierungen zur Anwendung kommen, müssen neben der DIN EN 14126 weiteren Vorgaben (ISO 16603, ASTM F 1670, ISO 16604, ASTM 1671, EN ISO 22610, ISO 22611, EN ISO 22612) entsprechen. Im Wesentlichen müssen sie die Körpervorderseite bedecken, langärmlig und flüssigkeitsdicht sein [15].

In der klinischen Praxis werden Einmalhandschuhe u. a. zum „Schutz des Trägers vor Kontamination mit Blut, Sekreten und Exkreten und zur Unterbrechung von Infektionsketten (…)“ eingesetzt. „Behandschuhte Hände sollten nur in speziellen Fällen desinfiziert werden, z. B. in Situationen, in denen ein häufiger Handschuhwechsel erforderlich, aber erfahrungsgemäß schwierig realisierbar ist bzw. der Wechsel zu einer Unterbrechung des Arbeitsflusses führt“ [30]. Die TRBA 250 legt unter 4.2.8 die Kriterien für Schutzhandschuhe fest [2]: „Als Handschuhe sind geeignet [22] – flüssigkeitsdichte, ungepuderte und allergenarme medizinische Handschuhe [22] mit einem Qualitätskriterium AQL (Accepted Quality Level) von ≤1,5 bei möglichem Kontakt zu Körperflüssigkeiten und -ausscheidungen; – flüssigkeitsdichte, ungepuderte, allergenarme und zusätzlich reinigungs- bzw. desinfektionsmittelbeständige Schutzhandschuhe [23] mit verlängertem Schaft zum Umstülpen bei Reinigungs- und Desinfektionsarbeiten, damit das Zurücklaufen der kontaminierten Reinigungsflüssigkeit unter den Handschuh verhindert wird“.

Vor dem Ausziehen der Handschuhe kann zusätzlich eine Desinfektion erfolgen, um eine Hautkontamination zu verhindern. Dieses Vorgehen und die Verwendung von 2 Paar Einmalhandschuhen sollten mit der jeweiligen Krankenhaushygiene abgesprochen sein.

Neben der allgemeinen Gefährdungsbeurteilung ist zusätzlich auch eine Gefährdungsbeurteilung psychischer Belastung als sinnvoll anzusehen (BGW 08-00-042) [7].

Bei Versicherten im Gesundheitswesen und vergleichbaren Tätigkeiten (z. B. Labor) bei einer Infektion mit SARS-CoV‑2 (COVID-19-Erkrankung) kommt die Anerkennung als Berufskrankheit 3101 in Betracht. Bei begründetem Verdacht zwischen der beruflichen Tätigkeit und dem Auftreten einer SARS-CoV-2-Infektion mit entsprechenden Krankheitszeichen, einer positiven SARS-CoV-2-PCR muss unverzüglich eine Anzeige als Berufskrankheit (BK 3101 der Anlage 1, DGUV) gemeldet werden. Diese kann durch einen Arzt oder den Unternehmer erfolgen [16, 17, 19].

Der gesetzliche Versicherungsschutz bleibt auch bestehen, wenn die Ausstattung mit der notwendigen Schutzausrüstung durch den Arbeitgeber nicht mehr gewährleistet werden kann. Grundsätzlich muss der Arbeitgeber den Arbeitsschutz sicherstellen und ggf. die gefährdenden Tätigkeiten einstellen. Der Versicherungsschutz bleibt auch bestehen, wenn der Arbeitnehmer die relevanten Vorgaben und Regeln nicht beachtet (Sozialgesetzbuch – SGB VII) [6, 13].

## Fazit für die Praxis

Das Regelwerk bezüglich der Arbeitssicherheit beim Umgang mit biologischen Arbeitsstoffen ist komplex. Dennoch dürfen die Vorschriften in der aktuellen Situation nicht vernachlässigt werden. Die maximale Tragezeit von FFP-Masken sollte eingehalten werden. Eine Aktualisierung der Gefährdungsbeurteilung – unter Einbeziehung der jeweilig zuständigen Fachärzte für Arbeitsmedizin, Fachkräfte für Arbeitssicherheit und unter Mitwirkung der Sicherheitsbeauftragten – ist notwendig (BGW Arbeitshilfen Gefährdungsbeurteilung).
